# Mindfulness Training in Primary Schools Decreases Negative Affect and Increases Meta-Cognition in Children

**DOI:** 10.3389/fpsyg.2015.02025

**Published:** 2016-01-12

**Authors:** Charlotte E. Vickery, Dusana Dorjee

**Affiliations:** ^1^School of Psychology, Bangor UniversityBangor, UK; ^2^Centre for Mindfulness Research and Practice, School of Psychology, Bangor UniversityBangor, UK

**Keywords:** mindfulness for children, mindfulness in school, emotional well-being, early intervention, meta-cognition, negative affect, emotion-regulation

## Abstract

Studies investigating the feasibility and impact of mindfulness programs on emotional well-being when delivered by school teachers in pre-adolescence are scarce. This study reports the findings of a controlled feasibility pilot which assessed acceptability and emotional well-being outcomes of an 8-week mindfulness program (Paws b) for children aged 7–9 years. The program was delivered by school teachers within a regular school curriculum. Emotional well-being was measured using self-report questionnaires at baseline, post-training and 3 months follow-up, and informant reports were collected at baseline and follow-up. Seventy one participants aged 7–9 years were recruited from three primary schools in the UK (training group *n* = 33; control group *n* = 38). Acceptability of the program was high with 76% of children in the training group reporting ‘liking’ practicing mindfulness at school, with a strong link to wanting to continue practicing mindfulness at school (*p* < 0.001). Self-report comparisons revealed that relative to controls, the training group showed significant decreases in negative affect at follow-up, with a large effect size (*p* = 0.010, *d* = 0.84). Teacher reports (but not parental ratings) of meta-cognition also showed significant improvements at follow-up with a large effect size (*p* = 0.002, *d* = 1.08). Additionally, significant negative correlations were found between changes in mindfulness and emotion regulation scores from baseline to post-training (*p* = 0.038) and baseline to follow-up (*p* = 0.033). Findings from this study provide initial evidence that the Paws b program in children aged 7–9 years (a) can be feasibly delivered by primary school teachers as part of the regular curriculum, (b) is acceptable to the majority of children, and (c) may significantly decrease negative affect and improve meta-cognition.

## Introduction

Research has robustly demonstrated that children’s psychological, emotional, and social well-being influences their future physical and mental health, educational outcomes, social prospects, and quality of life in adulthood (Currie et al., 2002). Specifically, the ability to regulate emotions is correlated with higher levels of well-being and learning outcomes in both children and adults (Barnes et al., 2003; Mendelson et al., 2010; Weare and Nind, 2011). The presence of effective coping and emotion regulation skills in early life protects psychological well-being and promotes resilience across the life span (Greenberg et al., 2003; Greenberg and Harris, 2012). Consequently, research in children over the last decade has expanded the focus from treatment and repairing of problems to investigating protective aspects of psychological functioning (Seligman and Csikszentimihalyi, 2000) involving early and preventive approaches (Diener and Seligman, 2002).

Promoting emotional well-being in schools is strongly advocated in the UK government policy through the national curriculum, and there is a ‘drive’ in education for evidence-based programs that consider the ‘whole child’ (Department for Education, 2014). There is a broad agreement among educators, policymakers, and the public that educational systems should support children to develop emotional and social skills in order to nurture positive health behaviors, meaningful relationships and become emotionally and socially responsible adults (Durlak et al., 2011; Weare, 2013). Arguably, the school setting offers a ‘gateway’ to provide early intervention and preventive programs (EIPPs) for children that enhance emotional, behavioral, and social learning opportunities (Rempel, 2012; Weare, 2013) and promote positive psychological functioning and emotion regulation capacities (Durak et al., 1997; Diener and Seligman, 2002).

The integration of EIPPs into the school context and curricula offers inclusive and equal opportunities for all children to learn ‘life skills’ which promote psychological well-being (Greenberg et al., 2004). However, given the growing demands on schools, it is important that research identifies curricular programs which offer sustainable and cost effective EIPPs which lead to positive long term well-being outcomes for children. Strategically drawing on the skills of classroom teachers to deliver well-being programs may be particularly relevant in this context. Therefore, the aim of the current study was to evaluate the impact of a mindfulness program (Paws b) delivered by classroom teachers to primary aged children as part of the Personal and Social Education (PSE) curriculum. Emotional well-being was evaluated across a number of domains including: mindfulness, emotion regulation, affect, positivity, and meta-cognition.

Mindfulness based interventions have been linked with enhancements in well-being and self-regulation across a broad range of research and theory (Kabat-Zinn, 2003; Singh et al., 2003; Blair and Diamond, 2008; Tang et al., 2012) and may offer a suitable EIPP for children. Mindfulness has been defined as a mental state or trait that can be developed and nurtured (Schonert-Reichl et al., 2015). It is understood as a dynamic process involving the intentional focus of the mind’s attention on thoughts, feelings, sensations and perceptions, and the ability to be aware of and connect with these experiences in a non-judgmental way (Kabat-Zinn, 1994). Mindfulness promotes the cultivation of a less automatic mode of mind, enhances awareness of internal processes and reduces reactive patterns of thinking, feeling and behaving (Chapman et al., 2013). Although the mechanisms by which mindfulness exerts its effects on psychological well-being are not yet fully understood, there is evidence that mindfulness enhances cognitive control (Tang et al., 2012), which is closely related to emotion regulation (Ochsner et al., 2002; Teper and Inzlicht, 2013).

Brain imaging research in adults has shown that practicing mindfulness engages brain networks involved in executive functioning (EF) which underlie complex mental processes and cognitive control capacities such as focused attention, inhibition, perspective taking and decentring; all linked to emotion regulation processes (Hölzel et al., 2011; Tang et al., 2012). It appears that mindfulness may influence cognitive aspects of information processing by enhancing the more controlled top-down processes (e.g., attention and inhibition) whilst at the same time reducing or balancing automatic arousal and appraisal systems associated with bottom-up processing (arousal and affect; Zelazo and Lyons, 2012). This interrelationship between top-down and bottom-up processing strategies highlights the importance of evaluating the impact of mindfulness on outcomes associated with both cognitive control and affective aspects of self-regulation (Zelazo and Lyons, 2012).

Meta-cognition is implicated in top-down information processes and cognitive aspects of emotion regulation. The conscious regulation of EF involves meta-cognitive skills (Fernandez-Duque et al., 2000) which include reasoning, self-reflective learning and self-awareness (Hassed and Chambers, 2014). In children meta-cognitive skills play a significant role in the knowledge and control they have over their thinking and learning processes (Cross and Paris, 1988). Both meta-cognition and emotional well-being are highlighted as important learning themes within UK educational policy and curricula [i.e., PSE and educational policy (Department for Education, 2011)], yet there are very few evidence based school programs promoting meta-cognition which can be delivered by school teachers for younger children (Education Endowment Foundation, 2015). Mindfulness-based programs in schools can be very relevant here.

A number of meta-analyses have concluded that mindfulness is efficacious for adults, producing small to large effect sizes for clinical and general populations across a range of physical health, psychiatric, cognitive, stress related, emotional, behavioral, interpersonal, and well-being outcomes (Grossman et al., 2004; Chiesa and Serretti, 2010; Piet and Hougaard, 2011; Piet et al., 2012). Both the theory and empirical research in adults suggests that mindfulness enhances self-regulation (Hölzel et al., 2011; Tang et al., 2012) but compared to the evidence base for adults, mindfulness research in children and adolescents is in its early stages. However, the emerging findings show that mindfulness can promote a number of well-being related outcomes in children.

A meta-analysis of mindfulness interventions with youth aged 6–21 years (Zoogman et al., 2014; *k* = 20) found enhancements in well-being in children and adolescents to be superior over active controls, with effect sizes in the small to moderate range (*p* ≤ 0.0001) for a variety of outcomes (i.e., emotion and behavioral regulation, depressive and anxiety symptoms, stress, attention and cognitive functioning). A further meta-analysis by Zenner et al. (2014) specifically investigated school-based mindfulness interventions for children and adolescents (*k* = 24; *n* = 1348) and found a medium overall effect size (Hedges, 1981; Hedges’ *g* = 0.40) across domains of well-being (e.g., resilience, stress, coping, and emotional problems) with an additional large effect size for areas of cognitive performance such as attention, inhibition and grades (*g* = 0.80). Finally, a systematic review of mindfulness based interventions in school settings (Felver et al., 2015), highlighted a number of limitations in the current research base for children and mindfulness including a lack of diverse outcome measurements such as multi-method and informant approaches and controlled studies using a follow-up design.

Current research on mindfulness in schools for primary aged children is very limited. A systematic review by Felver et al. (2015) identified that the average age across 28 peer reviewed studies (*N* = 3414) was 12.3 years. In fact, only eight studies in this review were conducted with primary aged children, therefore the evidence base across developmental stages is not equally representative. Indeed, a previous systematic review and meta-analysis highlighted much less research in early childhood and primary aged children than in older children and adolescents (Zenner et al., 2014). Of the eight primary school studies in the Felver et al. (2015) review, each study used a different type of mindfulness training and delivery format; additionally, only three studies involved the classroom teacher leading the delivery of a mindfulness program (*N* = 364; Liehr and Diaz, 2010; Schonert-Reichl and Lawlor, 2010; Britton et al., 2014). Furthermore, all of these studies were conducted outside of the UK, none included follow-up measures and only two formally evaluated acceptability for a conscript group of children (Schonert-Reichl and Lawlor, 2010; Britton et al., 2014). Although the latter two studies had significant strengths concerning both using active controls and measuring affective and cognitive aspects of emotional well-being and regulation, only one involved randomization (Britton et al., 2014) and neither measured meta-cognition. Overall, the studies identified improvements in affective disturbance, suicidal ideation (Britton et al., 2014), optimism, attention, and EF (Schonert-Reichl and Weissberg, 2014).

Regarding the investigation of effects of mindfulness on meta-cognition in children, only two studies have explored this variable (Flook et al., 2010; Weijer-Bergsma et al., 2012) with both finding improvements at either post training (Flook et al., 2010) or follow-up (Weijer-Bergsma et al., 2012). However, only one of these studies was conducted in the primary school context and for a general population sample (Flook et al., 2010). This study found improvements in meta-cognition and overall global executive control at post training for children with lower levels of baseline functioning. Whilst this study had a number of strengths including a randomized controlled design, the generalization of findings is limited by a lack of follow-up design, the exclusive use of informant based measures, effects restricted to children with lower baseline functioning and an outside facilitator delivering the mindfulness training.

Overall, the lack of research on the impact of mindfulness training with primary school aged children on emotion regulation and meta-cognition greatly limits implications of findings, particularly in the conscript context with teachers delivering mindfulness training. In addition, further feasibility and acceptability data is required to better understand which programs and formats best fit delivery for primary aged children by classroom teachers. It is essential that future studies expand the quality of research in this field by implementing controlled designs with manualized programs and report the ‘dosage’ of practice in order to aid replication of findings and to identify if mindfulness programs induce significant effects over time (Brown et al., 2015). The limited UK based research for mindfulness and children means that there is a gap in knowledge of how school based mindfulness interventions impact children in school, how they are received by children and schools, and if cultural and pedagogical differences exist. Such research is essential to inform education and well-being policies. In addition to evaluating and analyzing acceptability data, a further aim of this study was to expand the knowledge base and quality of research about mindfulness training in primary schools by (a) using a follow-up controlled design, (b) selecting a broad range of non-clinical validated emotional well-being measures, (c) including measures of both cognitive control and affect, (d) combining self-reports from children with evaluations from parents and teachers for some measures, and (e) reporting on the format of the mindfulness program (e.g., frequency and dosage). The study addressed two research questions, (1) Does mindfulness training enhance domains of emotional well-being and meta-cognition of children aged 7–9 years at post and follow-up, compared to controls? (2) Are selected constructs of emotional well-being modulated differently by mindfulness training in children aged 7–9 years? We hypothesized the training group would experience improvements in emotional well-being and meta-cognition which will be maintained at follow-up, compared to controls. To our knowledge, this is the first study in the UK to investigate the impact of a curriculum based mindfulness intervention when taught by classroom teachers to a conscript group of primary school children.

## Materials and Methods

The study received ethical approval from the Ethics Committee of the School of Psychology, Bangor University, prior to commencing. Informed consent was obtained from parents of all child participants before evaluation. Children were also asked individually whether they would like to participate before the start of each evaluation session and their decisions were respected. Informed consent was also obtained from parents and teachers providing informant assessments of children’s meta-cognitive abilities.

The feasibility pilot study followed a non-randomized wait-list controlled design and was part of a larger project that piloted and evaluated an implementation model involving training school teachers in mindfulness. Provided that participating teachers developed sufficient personal mindfulness practice, they were then trained in the Paws b mindfulness curriculum and the delivery to pupils.

### Participants

Seventy-one children (36 male), 7–9 years old (*M* = 7.90, *SD* = 0.64), were recruited from three primary schools in North Wales, matched on basic socio-economic characteristics. Allocation into the training and control groups was based on volunteer interest and availability of schools for implementation of the training and assessments with children. The first two schools to opt into the study were assigned to the training group (*n* = 33; 19 male, 14 female; age *M* = 8.00, *SD* = 0.66) and the last school to the control group (*n* = 38; 17 male, 21 female; age *M* = 7.82, *SD* = 0.61). None of the schools had previous experience with implementation of mindfulness programs into their curricula.

An independent samples *t*-test compared ages of children in the training group (*M* = 8.00, *SD* = 0.66) and the control group (*M* = 7.82, *SD* = 0.61) at baseline with no significant differences found *t*(69) = –1.22, *p* = 0.23, *d* = –0.30. A Pearson Chi square test also found no significant differences for gender between the training and control groups: *x*(1) = 1.17, *p* = 0.34.

All parents of participating children were asked to fill in the Behavior Rating Inventory of Executive Function (BRIEF; Gioia et al., 2000) measure and teachers were also asked to complete this measure for children whose parents consented for them to participate in the study. In the training group, teachers completed the BRIEF measure for 17 pupils before the intervention whilst only 16 measures on pupils were completed by teachers for this measure at follow-up. Of the 26 parents who completed the BRIEF measure before the start of the intervention program, only 17 also completed this measure at follow-up. For the control group, teachers completed the BRIEF measure for 20 pupils both at baseline and at follow-up. For the 22 parents in the control group who completed the BRIEF at baseline, only 13 also completed this measure at follow-up. Only measures from teachers (Te) and parents (Pa) completed at both pre and follow-up for participants in the training group (Te, *n* = 16, *M* = 7.75, *SD* = 0.58, 10 male; Pa, *n* = 17, *M* = 8.18, *SD* = 0.64, 11 male; overlap in same participants evaluated by Te and Pa, *n* = 8) and control group (Te, *n* = 20, *M* = 8.20, *SD* = 0.41, nine male; Pa, *n* = 13, *M* = 8.15, *SD* = 0.69, eight male; overlap in same participants evaluated by Te and Pa, *n* = 10) were included. See **Table 1** for detailed information of sample demographics.

**Table 1:** Demographic information for sample.

Variable	Paws b	EAU	Total/Average
Participants (*N*)	33	38	71
Age (years)			
*M*	8.00	7.82	7.90
*SD*	0.661	0.601	0.631
Gender (%)			
Female	42%	55%	49%
Male	58%	45%	51%

*Paws b, Mindfulness Program – training group; EAU, Education as normal – control group.*

### Measures

Child participants completed a brief demographics questionnaire at baseline and four self-report questionnaires at baseline, post-treatment and 3 months follow-up. An additional questionnaire measuring meta-cognition was completed by each participant’s school teacher and a parent/guardian at baseline and 3 months follow-up (post evaluation was not administered because the time criteria for the measure extended 6 months). Finally, a brief acceptability measure (adapted for age) was administered to the training group post program and provided an evaluation of participants’ experiences of the program.

#### Child Adolescent Mindfulness Measure (CAMM, Greco et al., 2011)

The CAMM is a ten item self-report measure for children 10 years and over. It measures mindfulness by assessing the degree to which children act with awareness, and observe and accept internal experiences with non-judgmental and non-avoidant responses. Respondents are asked to indicate how well each item reflects their experience over the last 2 weeks (e.g., ‘I keep myself busy so I don’t notice my thoughts and feelings’). The CAMM is a 6-point Likert scale ranging from (0) ‘never true’ to (5) ‘always true.’ Greco et al. (2011) recommend that scores are totalled and reversed so that higher scores indicate greater mindfulness. This measure has shown good internal consistency and concurrent validity, and small-moderate negative correlations with somatic complaints, internalizing symptoms, externalizing behavior problems, thought suppression and psychological inflexibility (*n* = 319, Cronbach’s α = 0.87; Greco et al., 2011).

#### Emotion Expression Scale for Children (EESC, Penza-Clyve and Zeman, 2002)

The EESC is a 16 item self-report measure of intrinsic and extrinsic emotion expression used for children between 8 and 12 years. The scale assesses emotional awareness and reluctance to express emotions (e.g., ‘I have feelings I can’t figure out’ and ‘I prefer to keep my feelings to myself’). The measure uses a 6-point Likert scale ranging from (0) ‘not at all true’ to (5) ‘extremely true.’ The EESC produces two subscales (emotional awareness and emotional expression) and a total score is calculated by summing the 16 item responses. Higher scores indicate poor emotional awareness and a greater reluctance to express emotion (Penza-Clyve and Zeman, 2002). This measure has shown high internal consistency and moderate test–retest reliability for the poor awareness and expressive reluctance factors, respectively (*n* = 208, Cronbach’s α = 0.83, *r* = 0.59; Cronbach’s α = 0.81; *r* = 0.56; Penza-Clyve and Zeman, 2002).

#### Sterling Children’s Well-being Scale (SCWBS, Liddle and Carter, 2010)

The SCWBS is a 12 item self-report for measuring positive aspects of emotional and psychological well-being (e.g., optimism, relaxation, and interpersonal aspects) in children 8–15 years. Six items measure ‘positive emotional state’ (PeS) and six ‘positive outlook’ (PO). Three additional items form a social desirability sub-scale assessing biased responding, and although they were included for complete data collection in this study, the scores were excluded from scoring. Respondents indicate how often each statement reflects their experience over the last 2 weeks (e.g., ‘I feel I’m good at some things’). The measure uses a 6-point Likert scale ranging from (0) ‘never’ to (5) ‘all of the time.’ The two scales of relevance to our study (PeS and PO) are computed from the 12 item responses; higher scores indicate a stronger PeS and PO and greater well-being (Liddle and Carter, 2010). The SCWBS has shown good internal and external reliability using a test–retest method (*n* = 701, Cronbach’s α = 0.83; *r* = 0.75, *p* < 0.01; Liddle and Carter, 2010). The SCWBS also has a strong positive correlation with the WHO-5 Quality of Life Well-being Index (Bech, 2004) which supports concurrent validity (*r* = 0.70, Liddle and Carter, 2010).

#### The Positive and Negative Affect Scale for Children (PANAS-C; Ebesutani et al., 2012, Adapted from Laurent et al., 1999)

The PANAS-C-short has been created as a shortened version of the PANAS-C (Laurent et al., 1999). The scale assesses positive and negative emotional expressiveness in children aged 6–18 years using 10 items. Five items relate to positive affect (PA) and five to negative affect (NA). Respondents indicate how often each statement (e.g., ‘Happy’ or ‘Afraid’) reflects their experience over the last few weeks. The measure uses a 6-point Likert scale ranging from (0) ‘very slightly or not at all’ to (5) ‘Extremely.’ Scores are summed for each factor (i.e., PA and NA) producing two total scores; higher scores for PA indicate greater experiences of PA and higher scores for NA indicate greater NA (Ebesutani et al., 2012). Ebesutani et al. (2012) reported a high internal consistency for the PANAS-C short NA factor and a moderate internal consistency for the PA factor, respectively. The 5-item PA could also discriminate mood disorders from an externalizing disorder (*n* = 799, Cronbach’s α = 0.86; *r* = 0.55) whilst the NA scale could discriminate between mood and non-mood disorders which supported discriminant validity *(n* = 799, Cronbach’s α = 0.82; *r* = 0.47; Ebesutani et al., 2012).

#### Behavior Rating Inventory of Executive Function – Teacher and Parent Versions (BRIEF-T and BRIEF-P; Gioia et al., 2000)

The BRIEF assesses EF behaviors that are linked to cognition, emotion, and behavior in children aged 5–18 years; it is designed to be completed by parents and or teachers. For the purpose of this study, both parents and teachers completed one of the core scales of the BRIEF (meta-cognition) which included 44 items made up of five indices: Initiate, Working Memory, Plan/Organize, Organization of Materials, and Monitor. For each participant, a parent/guardian and a teacher were invited to respond to items relating to EF behavior over the last 6 months using a 3-point Likert scale (‘never,’ ‘sometimes,’ ‘often’). Scores are summed and higher scores indicate higher levels of executive dysfunction associated with poorer meta-cognition (Gioia et al., 2000). Both the BRIEF-P and BRIEF-T demonstrated high internal consistency (Cronbach α = 0.80; α = 0.98) and good test–retest reliability, respectively (*n* = 1,419, *r* = 0.82 teachers: *n* = 720, *r* = 0.88; Gioia et al., 2000).

#### Acceptability Measure

An acceptability questionnaire was partly informed by previous acceptability questions evaluating a school based mindfulness program (Kuyken et al., 2013). The questionnaire included four brief questions, suitable for 7–9 year olds, to evaluate participant experiences of practicing mindfulness in school. Question (1) asked participants if they liked doing mindfulness at school and used a Likert scale of 1–7 in schematic faces ranging from unhappy to very happy. Question (2) asked how often participants practiced mindfulness outside of school using a 4-point Likert scale (never, rarely, often, every day). Question (3) asked participants if they would like to carry on practicing mindfulness in school, with three answers (Yes, No, and Maybe). Question (4) invited participants to describe what they liked/disliked about practicing mindfulness. This measure was administered post program to the training group only.

### Procedure

Key Stage 2 [KS 2 (a stage of the state education system in England and Wales for 7–11 year olds)] from each school received a brief talk about mindfulness and their potential participation/involvement in the study. Children were then provided with sealed envelopes containing an information pack about the study to take home to their parents. The packs included: a parental information sheet, an age-adapted information sheet for the child and a parental/guardian consent form. An information session was also held for parents and children so questions and further information about the study could be clarified. Meetings were also held with participating teachers. For children whose parents did not provide consent, evaluation did not take place and all children participated in the Paws b program as part of the regular curriculum. Teachers in the training group volunteered to participate (*n* = 2) and were initially trained in a mindfulness course called .b Foundations (Mindfulness in Schools Project, 2015). Six months after this course, the teachers were assessed by an expert mindfulness trainer to ensure they had developed sufficient personal mindfulness practice, and were then trained in the delivery of the Paws b curriculum. Teachers from the control school (*n* = 3) were offered training in mindfulness and the Paws b curriculum at the end of the study.

#### Intervention (Paws b)

The Paws b program (Mindfulness in Schools Project, 2015) was developed and piloted by Sarah Silverton, Tabitha Sawyer, and Rhian Roxburgh at Ysgol Pen Y Bryn in collaboration with the Mindfulness in Schools Project and based on good practice programs such as Susan Kaiser-Greenland’s (USA), and the Mindfulness in Schools Project’s .b program for secondary schools. The intervention involved formal and informal mindfulness practices including a range of adapted mindfulness practices for children aged 7–11 years. The Paws b program was delivered by two school teachers in the training group across two schools for children in years 3 and 4 (i.e., 7–9 years). Teachers delivered the program as part of PSE lessons in the classroom setting to approximately 30 pupils. The Paws b program aimed to support children to develop more mindful and less automatic relating to their present moment experiences in the classroom. The six themes covered in the Paws b program (i.e., ‘Our Amazing Brain,’ ‘Puppy Training,’ ‘Finding a Steady Place,’ ‘Dealing with Difficulty,’ ‘ The Story Telling Mind,’ and ‘Growing Happiness’) can be flexibly delivered to suit school demands using 1 h or ½ h lessons (12 half hour lessons overall). In this study, two of the themes were covered over 2 weeks each whilst the remaining four were delivered in one session each as part of the PSE curriculum. Participants were also invited to do optional “Give it a Go” mindfulness tasks and home practice sheets. CDs or Mp3 recordings were not utilized for home practice to increase accessibility for children who might not be able to use/access equipment at home. Additionally, the teachers continued with approximately 5–10 min a week of informal mindfulness practice in school, between post and follow-up. This was the first time school teachers delivered the Paws b training to their pupils after training in the curriculum, and 7 months after they had completed the initial mindfulness training.

The delivery of the program within PSE allowed mindfulness to promote learning outcomes for PSE within ‘KS 2’ curriculum [i.e., access learning opportunities and experiences to support health and emotional well-being and promote physical and social awareness of learners (Department for Children, Education, Lifelong Learning, and Skills, 2008)]. The control group continued their usual PSE curriculum without the addition of mindfulness throughout the study period, and the teachers in the training group were encouraged to continue with informal mindfulness practices after the completion of the Paws b training.

#### Evaluation: Self-Report Measures for Children

The researcher was allocated a quiet room for small groups of children (maximum 10) to complete the four self-report measures at pre-training, post-training, and follow-up. At the beginning of each evaluation session, children confirmed their consent verbally, they were reminded that their answers were confidential and asked to respect each other’s privacy in completing the responses. Instructions and questions for each measure were read out by the researcher and also printed on each questionnaire; participants were encouraged to ask for help from the researcher if they had difficulty comprehending questions. Each questionnaire session lasted approximately 45 min, including a short break to support the participants’ focus and motivation. On completion of each evaluation session, a verbal de-briefing took place (e.g., what happens with the questionnaires, re-evaluation and answering participants’ questions). Participants received a small reward for their participation after each evaluation session (e.g., a colored pencil). At follow-up, participants received a final verbal debriefing about the hypotheses of the study.

#### Evaluation: Informant Based (Parent/Teacher)

Following pre and follow-up evaluation sessions with participants, each teacher, and parent of the child were provided with a sealed envelope that included the meta-cognition questionnaire, accompanying instructions, an information sheet, and a return envelope. Parents/guardians and teachers of each child were asked to complete the meta-cognition questionnaire and return it in a sealed envelope to the school.

### Data Analysis

All data was double entered and discrepancies followed up and clarified. Five children provided 50% or fewer responses (a pre-set cut-off point) on specific measures, which resulted in this data being removed and excluded from the analyses.

The four questions of the acceptability survey completed by the training group were presented using descriptive statistics and reported using percentages. All longitudinal comparison data was analyzed using the statistical software IBM SPSS Statistics version 20 (IBM Corp, 2011). Statistical significance levels (two-tailed) were set at *p* < 0.05. Effect sizes for ANOVAs are reported as eta-squared and Cohen’s *d* for *t*-tests (Cohen, 1988). For each of the four well-being measures, separate 2 (group) × 3 (time) mixed-factorial ANOVAs were conducted to examine changes between the two groups (training and control), over the three time points (pre-training, post-training, and 3-months follow-up). Significant interactions were further investigated using *t*-tests. For the meta-cognition measure a separate 2 × 2 × 2 mixed-factorial ANOVA was conducted to examine changes for the two groups (training and control), at two time-points (pre-training, and follow-up) and between the two raters (parent and teacher).

Finally, Pearson correlations were used to assess whether changes in mindfulness scores (CAMM) were associated with changes in well-being. To derive the change scores, difference scores for all well-being measures were calculated between post- and pre-training, and follow-up and pre-training time-points. The training and control groups were analyzed separately to determine whether correlations between variables were related to the intervention. Additionally, specific responses for the acceptability questionnaire were correlated with the above difference scores (i.e., question 1, “Did you like practicing mindfulness in school?” and question 4, “How often did you practice mindfulness outside of school?”).

**Table 2** provides descriptive statistics for the complete data set on each emotional well-being and meta-cognition measure at pre-, post-, and follow-up time points.

**Table 2:** Descriptive statistics for emotional well-being and meta-cognition measures at pre-, post-, and follow-up.

	Paws b									EAU								
	Pre			Post			Follow-up			Pre			Post			Follow-up		
Measure	*n*	*M*	*SD*	*n*	*M*	*SD*	*n*	*M*	*SD*	*n*	*M*	*SD*	*n*	*M*	*SD*	*n*	*M*	*SD*
CAMM	34	22.20	6.33	34	24.10	5.51	34	27.80	5.00	30	23.62	6.92	30	22.94	6.62	30	26.12	6.98
EESC:PEA	35	19.34	7.01	35	19.39	6.94	35	17.16	6.43	31	18.59	7.44	31	19.80	5.60	31	19.57	7.97
EECS:ER	35	25.50	6.12	35	22.65	6.16	35	19.50	5.49	31	21.10	7.36	31	22.10	5.80	31	19.50	5.49
SCWBS:PeS	35	20.12	4.74	35	19.95	5.10	35	19.83	5.85	31	18.55	5.20	31	20.81	5.45	31	18.37	5.40
SCWBS:PO	35	21.76	3.87	35	20.45	5.27	35	20.31	5.70	31	19.25	5.35	31	20.52	4.95	31	18.70	5.34
PANAS-C:PA	34	16.78	4.31	34	17.39	4.12	34	16.74	4.95	31	17.47	6.00	31	19.12	4.72	31	17.00	5.10
PANAS-C:NA	34	10.26	3.58	34	10.29	3.81	34	8.85	3.07	31	10.57	3.21	31	10.29	3.81	31	12.14	4.62
BRIEF:T	16	69.13	27.48	–	–	–	16	52.44	17.93	20	73.55	21.75	–	–	–	20	67.10	26.10
BRIEF:P	17	70.71	12.14	–	–	–	17	77.88	17.18	13	74.69	16.64	–	–	–	13	66.00	20.46

*Paws b, training condition; EAU, education as normal; *n*, number of participants; *M*, mean score; *SD*, standard deviation; CAMM, Child and Adolescent Mindfulness Measures; EESC:PEA, Emotion Expression Scale for Children: Positive Emotional Awareness; EESC:ER, Emotion Expression Scale for Children: Emotional Reluctance; SCWBS:PeS, Stirling Children’s Well-being Scale: Positive Emotional Expression; SCWBS:PO, Stirling Children’s Well-being Scale: Positive Outlook; PANAS-C: PA, The Positive and Negative Affect Scale for Children: Positive Affect; PANAS-C:NA, The Positive and Negative Affect Scale for Children: Negative Affect; BRIEF:T, Behavior Rating Inventory of Executive Function-Teacher; BRIEF:P, Behavior Rating Inventory of Executive Function-Parent.*

## Results

### Acceptability

The majority of participants in the training group (76%) reported ‘liking,’ ‘liking a lot,’ or ‘extremely liking’ practicing mindfulness at school. The remaining 18.1% reported either ‘extremely disliking’ or ‘ disliking’ practicing mindfulness at school and 6.1% reported that they ‘didn’t mind’ practicing mindfulness at school. When participants were asked if they would like to carry on doing mindfulness at school, 61% responded ‘yes,’ 33% ‘maybe,’ and 6.1% responded ‘no.’ The most popular descriptions for what was liked about practicing mindfulness included ‘watching videos’ (24%) and ‘feeling calm’ (21%). The most disliked aspects of practicing mindfulness were reported as ‘nothing,’ meaning there wasn’t anything they did not like (69.7%), followed by a specific Paws b practice known as ‘Feet On Floor, Bottom/Bum/Body On Chair’ (FOFBOC; 15.1%) – a grounding practice used for responding to emotional difficulty and involving focused attention on the lower body. Finally, participants were asked how frequently they practiced mindfulness outside of school for which 21.2% responded ‘never,’ 39.4% ‘rarely,’ 30.3% ‘often,’ and 9.1% ‘everyday.’ Correlational analysis explored this acceptability data further. There was no significant correlation between the degree to which children reported liking practicing mindfulness and how often they practiced outside school, [*r*(33) = 0.25, *p* = 0.16]. However, a significant correlation was found between the degree to which children liked practicing mindfulness and whether they wanted to continue practicing mindfulness at school, [*r*(33) = 0.70, *p* < 0.001].

### CAMM

A 2 × 3 mixed-factorial ANOVA showed no main effect of group *F*(1,62) = 0.16, *p* = 0.69, η^2^ = 0.003 and no time by group interaction *F*(2,124) = 1.53, *p* = 0.22, η^2^ = 0.024, but a significant effect of time was found, *F*(2,124) = 10.75, *p* ≤ 0.001, η^2^ = 0.14. There was no overall change in CAMM scores from pre- to post-training *t*(66) = –0.62, *p* = 0.54, but a significant increase from post-training to follow-up was found *t*(63) = –3.70, *p* < 0.001, *d* = –0.46. In the absence of an interaction, it was investigated whether there were any indications of increases in mindfulness being related to improvements in well-being in the training group. The correlational findings showed that changes in CAMM scores between pre- and post-training were significantly and negatively correlated with changes in EESC from pre- to post-training (*r* = –0.38, *p* = 0.038), and similarly, changes in CAMM between pre-training and follow-up were significantly and negatively correlated with changes in EESC between pre-training and follow-up (*r* = –0.40, *p* = 0.033). Both of these correlation analyses were non-significant for the control group, respectively (*r* = –0.14, *p* = 0.42, and *r* = –0.16, *p* = 0.37). There were no other significant correlations between changes in mindfulness and changes in other measures within the training group only. The internal consistency of the scale was in the poor to questionable range at pre-, post- and follow-up, Cronbach’s α = 0.54, 0.60, and 0.68, respectively.

### EESC

A 2 × 3 mixed-factorial ANOVA for the emotional awareness scores showed no significant main effects of group *F*(1,64) = 0.29, *p* = 0.59, η^2^ = 0.005 or time *F*(2,128) = 0.76, *p* = 0.47, η^2^ = 0.012 and no significant time by group interaction *F*(2,128) = 1.30, *p* = 0.28, η^2^ = 0.020. Similarly, a 2 × 3 mixed-factorial ANOVA for expressive reluctance reported no significant main effects of group *F*(1,64) = 0.02, *p* = 0.90, η^2^ < 0.001 or time *F*(1.81,115.61) = 1.66, *p* = 0.20, η^2^ = 0.025 and no significant time by group interaction *F*(1.18,115.61) = 2.21, *p* = 0.12, η^2^ = 0.033. The internal consistency of the scale was in the questionable to good range at pre-, post-, and follow-up for poor emotional awareness, Cronbach’s α = 0.79, 0.66, and 0.81, respectively. The internal consistency of the scale was in the poor to acceptable range at pre-, post-, and follow-up for expressive reluctance, Cronbach’s α = 0.73, 0.55, and 0.63, respectively.

### SCWBS

A 2 × 3 mixed-factorial ANOVA for PeS showed no significant main effects of group *F*(1,64) = 0.47, *p* = 0.50, η^2^ = 0.007, or time *F*(2,128) = 2.14, *p* = 0.12, η^2^ = 0.032, and no significant time by group interaction *F*(2,128) = 2.16, *p* = 0.20, η^2^ = 0.033. For PO a 2 × 3 mixed-factorial ANOVA reported no significant main effects of time *F*(2,128) = 1.26, *p* = 0.29, η^2^ = 0.019, or group *F*(1,64) = 2.20, *p* = 0.14, η^2^ = 0.033, and no significant interaction *F*(2,128) = 1.65, *p* = 0.20, η^2^ = 0.025. The internal consistency of the scale was in the acceptable to good range at pre-, post-, and follow-up for PeS with Cronbach’s α = 0.76, 0.79, and 0.82, respectively. The internal consistency of the scale was in the questionable to acceptable range at pre-, post-, and follow-up for PO Cronbach’s α = 0.65, 0.78, and 0.76, respectively.

### PANAS-C

**FIGURE 1:**
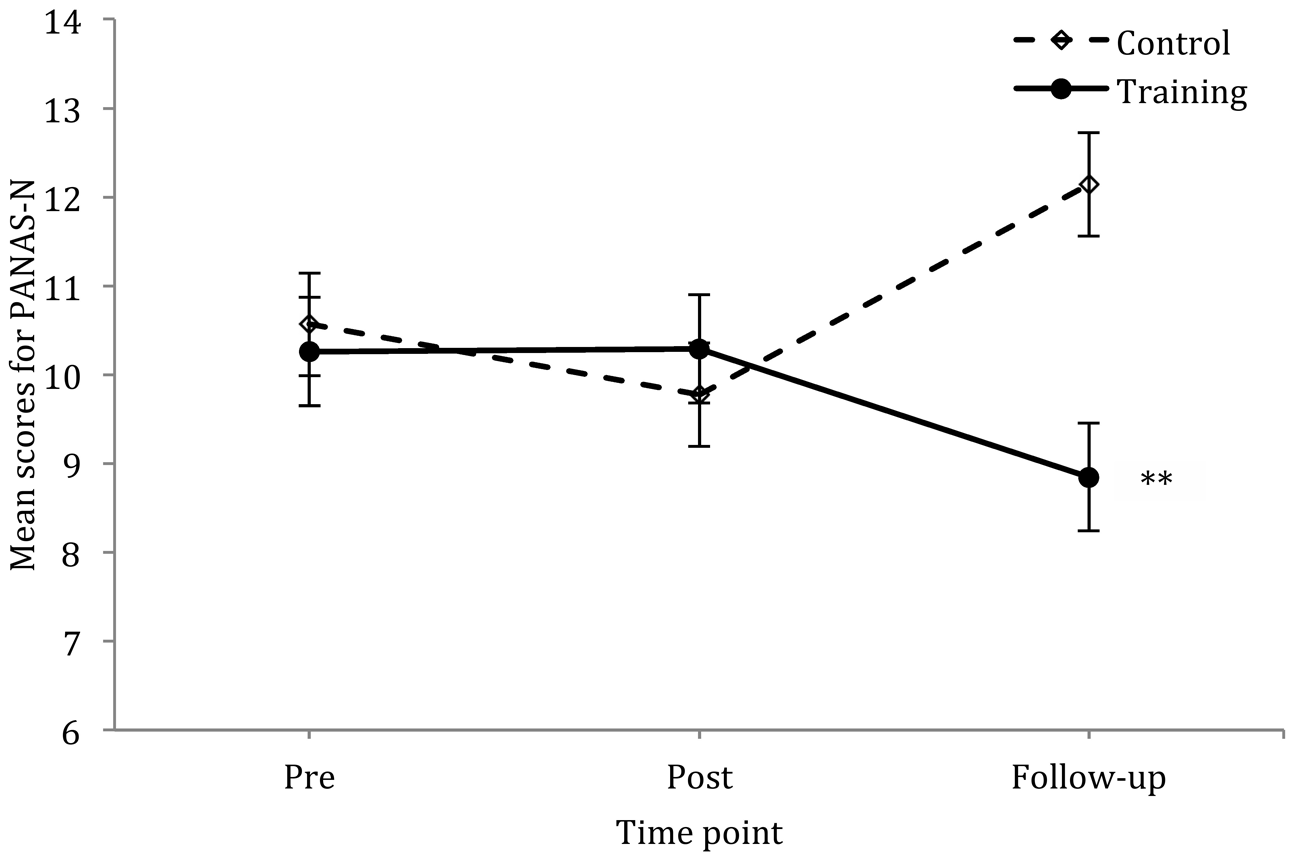
**Mean scores for PANAS-N across three time points for the control and training group; vertical lines depict standard errors of the means.** ^∗∗^*p* < 0.01.

A 2 × 3 mixed-factorial ANOVA for NA scores reported no main effects of group *F*(1,63) = 2.62, *p* = 0.11, η^2^ = 0.040, or time *F*(2,126) = 0.37, *p* = 0.69, η^2^ = 0.006 but there was a significant time by group interaction *F*(2,126) = 6.13, *p* = 0.003, η^2^= 0.089. Follow-up *t*-tests showed that while at baseline there was no difference between the two groups *t*(68) = 0.68, *p* = 0.50, *d* = 0.16, the training group showed lower NA scores than the control group at follow-up *t*(64) = 3.40, *p* = 0.001, *d* = 0.84. No significant between group difference was found at post-test. A 2 × 3 mixed-factorial ANOVA (as above) for PA showed no significant main effects of group *F*(1,63) = 1.05, *p* = 0.31, η^2^ = 0.016, or time *F*(1.78,112.21) = 1.98, *p* = 0.15, η^2^ = 0.030, and no significant time by group interaction *F*(1.78,112.21) = 0.53, *p* = 0.57, η^2^ = 0.008. See **Figure 1** for mean scores of PANAS-N across pre-, post-, and follow-up time points in the training and control group. The internal consistency of the scale was in the unacceptable to questionable range at pre-, post-, and follow-up for NA, Cronbach’s α = 0.41, 0.55, and 0.66, respectively. The internal consistency of the scale was in the questionable to good range at pre-, post-, and follow-up for PA, Cronbach’s α = 0.80, 0.68, and 0.73, respectively.

### BRIEF

A 2 × 2 × 2 mixed-factorial ANOVA with factors of time (pre-training and follow-up), group (training and control), and raters (teachers and parents) showed a significant main effect of time *F*(1,62) = 6.29, *p* = 0.02, η^2^= 0.078, non-significant main effect of group *F*(1,62) = 0.38, *p* = 0.54, η^2^= 0.006, and a non-significant main effect of rater *F*(1,62) = 2.21, *p* = 0.14, η^2^= 0.034. A significant time by rater interaction was found *F*(1,62) = 4.83, *p* = 0.03, η^2^= 0.060, and time by group by rater interaction *F*(1,62) = 7.05, *p* = 0.01, η^2^= 0.088. The time by group interaction was not significant *F*(1,62) = 0.33, *p* = 0.57, η^2^= 0.005.

The significant time and time by group by rater interaction was followed up with *t*-tests comparing the teacher and parent ratings for the control and training groups at baseline versus follow-up. The teachers ratings of the control group did not change over time *t*(19) = 1.31, *p* = 0.21, *d* = 0.30, but the training group teacher ratings decreased significantly from baseline to follow-up *t*(15) = 3.74, *p* = 0.002, *d* = 1.08, showing an improvement in meta-cognition. Similarly, the parents ratings of the control group did not change between baseline and follow-up *t*(12) = 1.24, *p* = 0.24, *d* = 0.35. For the training group, the parents ratings showed a slight increase for BRIEF scores from baseline to follow-up *t*(16) = –2.36, *p* = 0.03, *d* = –0.61. The internal consistency of the scale was in the excellent range at pre and follow-up for teachers, Cronbach’s α = 0.98 and 0.99, respectively. The internal consistency of the scale was in the excellent range at pre- and follow-up for parents, Cronbach’s α = 0.94 and 0.96, respectively.

**FIGURE 2:**
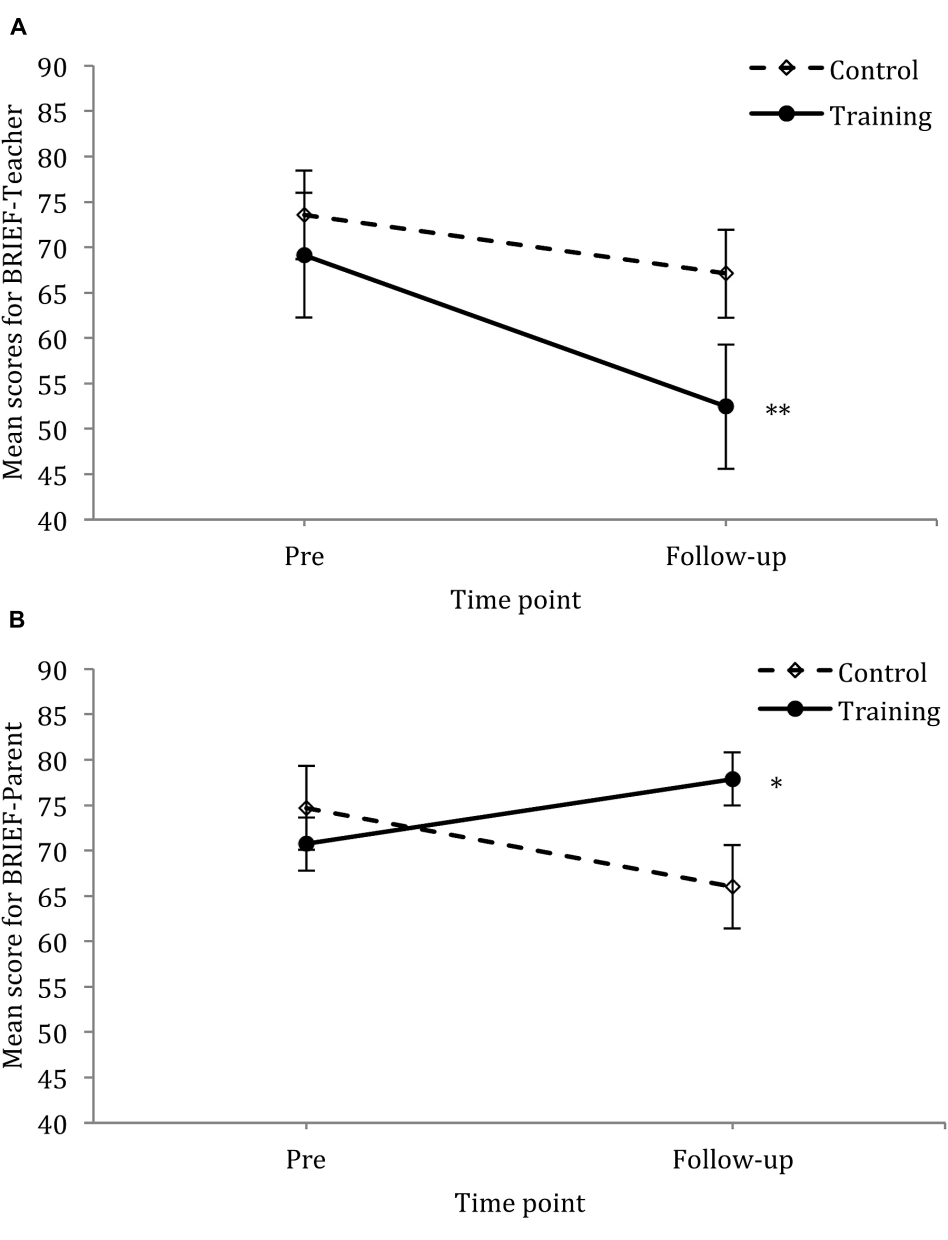
**Mean scores for the BRIEF-T **(A)** and BRIEF-P **(B)** across two time points using data which included both pre- and follow-up scores only; vertical lines depict standard errors of the means.** ^∗^*p* < 0.05, ^∗∗^*p* < 0.01.

See **Figure 2** for mean scores of the BRIEF-T and BRIEF-P across post- and follow-up time points for the control and training group.

## Discussion

This study provides initial evidence that the Paws b mindfulness program can be feasibly delivered to a conscript group of school children aged 7–9 years by their classroom teachers within the PSE curriculum and in a UK setting. Our findings show that the Paws b program was acceptable to the majority of children in this study, and suggest that it significantly decreased NA. We also found significant improvements in meta-cognition at follow-up in teacher ratings of children. We did not find significant longitudinal changes in measures of mindfulness, PA, emotional awareness and expressive reluctance, and positive well-being. However, significant negative correlations between mindfulness scores (CAMM) and poor emotional awareness, together with a greater reluctance to express emotion (EESC) for baseline to post-training and baseline to follow-up, suggested tentative improvements in emotional well-being. These findings suggest that the Paws b mindfulness program enhances specific domains of emotional well-being for children aged 7–9 years compared to controls at follow-up and that selected constructs of emotional well-being respond differently to mindfulness training for children aged 7–9 years.

Significant findings with large effect sizes for NA and meta-cognition at follow-up are very promising in the context of a brief intervention with a modest ‘dosage’ of mindfulness practice, the first time classroom teachers delivered mindfulness training to their pupils after being trained in mindfulness 6 months earlier, and given that the majority of children did not engage in extra mindfulness practice outside of the school program. Most previous studies on mindfulness in schools have involved experienced mindfulness trainers or school teachers with considerable mindfulness practice experience. This makes our study more naturalistic by reflecting realistic challenges to implementing mindfulness in schools and possible outcomes. It is also important to acknowledge that the significant findings with a conscript class in this study were obtained using more stringent statistical analyses (did not include statistical adjustments for demographics and baseline levels of variables) which turned out insignificant in other studies (e.g., Flook et al., 2010; Huppert and Johnson, 2010; Kuyken et al., 2013).

The majority of pupils participating in the Paws b program liked practicing mindfulness at school (76%). Notably, this acceptability finding is superior to the average acceptability rating for new elements of the curriculum in primary aged children in the UK (50%; Department for Education, 2011). In addition, correlational analysis highlighted that although no significant correlation between the degree children reported liking practicing mindfulness and how often they practiced outside school was found, there was a significant relation between the degree children liked practicing mindfulness and whether they wanted to continue practicing mindfulness at school. The finding that the majority of children (60.6%) in this study reported never or rarely practicing mindfulness outside of school, replicates a finding by Huppert and Johnson (2010). Since pre-adolescent children have less developed EF abilities than older children and adults, the children in our study may have had difficulty with self-initiating and motivating themselves to engage with mindfulness independently, and this may provide an explanation for why the training group’s enjoyment of mindfulness and desire to continue with it in school did not automatically enhance their motivation to practice autonomously outside of school. It is likely that parental engagement and support will be important in encouraging home mindfulness practice, especially in younger age groups. This will depend on the parental groups involved and their willingness to support their children, and the children’s receptivity to parental involvement. Indeed, previous research has consistently reported associations between enhanced socio-emotional outcomes in children and parental support (El Nokali et al., 2010; Shucksmith et al., 2010). This finding points to the importance of identifying differences in how children might use mindfulness skills independently and across developmental stages. Such information would inform age appropriate ways of maximizing children’s engagement in mindfulness practice both within and outside of school, and with and without parental involvement.

Longitudinal comparisons of self-report data showed that pupils participating in the Paws b program reported significant improvements in NA with a large effect size at follow-up compared to the control group. Non-significant findings for NA at post training are consistent with the majority of existing studies evaluating this well-being variable in pre-adolescent children following a mindfulness intervention (i.e., Mendelson et al., 2010; Schonert-Reichl and Lawlor, 2010; Corbett, 2011). It is noteworthy that the only study to identify significant improvements in NA at post training in children to date (Broderick and Metz, 2009) included a sample of 16–19 year olds. Together, previous research and current findings may suggest a delayed manifestation of the benefits of mindfulness for NA in primary aged children, and this may provide an explanation for non-significant findings at post training in the majority of currently available studies. There is clearly a need for research to replicate this finding and to establish potential moderating factors (e.g., age and amount of mindfulness training), which might influence how children benefit and respond to mindfulness, to inform the development of effective mindfulness programs for primary school children.

Findings from this study also point to improvements in meta-cognition in children in the training group as rated by school teachers; however, the reliability of this finding should be considered cautiously because it was not supported by follow-up parental ratings of meta-cognition. Given that the same teachers delivering the mindfulness program rated meta-cognition in this study at pre- and follow-up, it is possible that demand characteristics may have encouraged biased responding by teachers. In contrast, it is also possible that differences found between teacher and parental meta-cognition ratings were in fact reliable and reflected the teacher’s ability to more readily identify changes in cognitive functioning than parents. Or that the findings in school related to a context effect in which learning did not generalize beyond the school environment. The discrepancy of teacher and parent findings may have also resulted from less than half of the sample (*n* = 8) being the same children evaluated by both teachers and parents in the training group (compared to 10 in the control group). Overall, the meta-cognition finding should not be overlooked and requires replication to ascertain its reliability.

Together, the significant findings for NA and meta-cognition at follow-up may highlight a possible mechanism responsible for the improvements in NA found in this study. Specifically, previous research has linked the control of thoughts and goals involving meta-cognitive skills to the alleviation of negative emotions (Davis et al., 2010; Raes and Williams, 2010). It is therefore possible that mindfulness training in this study improved the meta-cognitive skills of children which then enhanced self-regulation capacities and produced beneficial changes in NA. This would suggest that top-down mechanisms of information processing involving EF were involved in the beneficial effects observed.

Future research and implementation efforts would benefit from better understanding the impact and role mindfulness might have on different aspects of self-regulation in children (i.e., top-down and bottom-up information processing strategies), particularly in pre-adolescence which is a time of considerable EF development (Davis et al., 2010). Such findings would advance developmental psychological models of mindfulness and training programs, and our understanding of how mindfulness might also support education and learning. Programs which promote EF within the school curriculum may be very beneficial in supporting learning and well-being with limited demands on school resources. In building on the strengths of teachers to implement mindfulness programs in schools within the curriculum, children would receive equal and accessible opportunities that promote developmental success; further, teachers would gain strategies to promote important aspects of learning such as meta-cognition.

While this study did not find significant improvements in mindfulness in the training group, follow-up correlational analyses revealed patterns of improvement in emotional well-being between measures of mindfulness and emotional awareness and expression (i.e., CAMM and EESC) for pre- and post-training and pre-training and follow-up. Because these correlations were not found in the control group, this finding suggests that the children receiving the Paws b program showed a pattern of improvement across some domains of emotional well-being which might emerge as statistically significant in larger scale studies. Overall, Paws b shows promise as an accessible and a potentially cost effective mindfulness program that can be readily integrated into the PSE curriculum for KS 2 children.

In summary, this was the first study conducted in the UK reporting the delivery of a school based mindfulness program by classroom teachers for a conscript group of primary school aged children. It supports findings from three previous studies conducted outside of the UK which also demonstrated the feasibility of this method of delivery (Liehr and Diaz, 2010; Schonert-Reichl and Lawlor, 2010; Britton et al., 2014). The current study also expands the very limited research evaluating children’s acceptability of a school based mindfulness program delivered by their teachers, which can inform the future development of mindfulness programs (Schonert-Reichl and Lawlor, 2010; Britton et al., 2014). This study provides initial indicators of significant improvements in NA and meta-cognition at follow-up for primary aged children as a result of a mindfulness program at school. Fidelity of the Paws b program was enhanced by training the teachers in a standardized delivery format. Additionally, fidelity was enhanced by using a mindfulness teacher-trainer and a school teacher with experience in delivery of the Paws b curriculum to assess teacher’s mindfulness experience prior to curriculum training and to provide teaching feedback/supervision during the delivery of Paws b. Finally, the inclusion of a broad range of age appropriate measures of emotional well-being, rather than evaluations of symptomology of mental health (e.g., depression or anxiety symptoms) or problem behavior (e.g., aggression), possibly reduced the potential of ceiling or floor effects when these outcomes are measured in general populations.

### Clinical Implications

Given the established strong correlation between symptoms of anxiety and depression and NA in both adults and children, and NA being a general predictor of anxiety and depression (Watson et al., 1988; Barlow, 2000), the finding in this feasibility pilot study that mindfulness significantly reduced negative affect indicates the potential of mindfulness to promote resilience and protect psychological well-being in children aged 7–9 years. In the context of early and preventative approaches to well-being, mindfulness delivered in schools as part of the curriculum may offer rich opportunities for schools and teachers to nurture children’s skills and capacities in self-regulation, and reduce the risk of emotional problems manifesting. Consequently, mindfulness may offer an approach to promoting emotional well-being not only for normally developing children but also for those at risk of developing problems. Findings from the current study that mindfulness enhanced meta-cognition have potential implications for self-regulation, developmental success, and learning, not only for the purpose of education, but to prepare children for challenges involving further education and working life which require them to effectively problem solve, make sense of their experiences, reflect on and adapt their performance.

### Limitations and Future Directions

This study did not allow for randomization of groups and further research is needed using a more stringent design. In addition, this study did not utilize an alternative active control (in addition to treatment as usual control) which somewhat reduces the validity of conclusions concerning benefits of the mindfulness program on emotional well-being and effect sizes being attributable to mindfulness alone, or other non-specific intervention factors such as novelty. A further limitation of this study was the use of non-blind teacher and parental ratings which limited the reliability of findings for meta-cognition. However, the parallel use of both raters aimed to increase the internal validity of informant-based measures and enabled us to exercise caution in the interpretation of significant improvements for meta-cognition regarding discrepancies between teacher and parental ratings at follow-up. Although this study used a follow-up design, it was limited to 3 months.

It is noteworthy that in the absence of a validated mindfulness measure for children below the age of ten years, the ability of the CAMM to measure mindfulness in the current sample may have been limited. Future research will need to develop validated and reliable measures of mindfulness for younger children. Similarly, it is important to acknowledge that we have not found significant changes on three of the four measures with children and for some of the measures the reliability assessments were inadequate or poor. This includes the PANAS-C measure of NA where we have found significant changes at follow-up. The low reliability for some measures highlights questions about suitability of self-reports in research with children. It is therefore important that future research employs experimental measures and psycho-physiological assessments such as heart-rate variability and electroencephalography derived brain function indexes in addition to self-reports and informant-based measures to provide a more complete picture of well-being relevant changes with mindfulness training in children and adolescents.

A further consideration for future research concerns data collection in the school context given the importance of collecting data in a way that minimizes impact on the pupils and teachers daily education schedule and school routine. Therefore, many decisions concerning data collection in the current study were guided by the school context (e.g., days and time of data collection). Additionally, issues concerning room availability and time constraints within the school setting meant that all data at each time point was collected in small groups of children in a separate room to their classroom (∼8–10). It remains an open question whether data collection in smaller groups would have improved the reliability of some of the measures.

Future studies will need to investigate the long-term term sustainability of the benefits of Paws b which will ascertain if there is a need for continued mindfulness programs or ‘booster’ lessons within and across school years, as found in other evidenced based school programs for emotional well-being (e.g., FRIENDS; Briesch et al., 2010). Further studies will need to assess how conscript delivery of mindfulness training as part of the regular curriculum can benefit children with learning difficulties and disabilities for whom programs which promote EF could be extremely valuable. Research should also turn its attention to assessing diverse groups of teachers with varied mindfulness experience and control for potential moderating factors such as motivation, personality, empathy, and baseline levels of mindfulness. Finally, although we have postulated that significant decreases in NA at follow-up found in this study may have been mediated by significant improvements in meta-cognition, future studies will need to investigate the exact mechanism pathways responsible for improvements in emotional well-being in children as a result of mindfulness, and account for their developmental trajectories.

## Conclusion

This study showed that the Paws b mindfulness program delivered by classroom teachers significantly reduced NA and enhanced meta-cognition in children aged 7–9 years at 3 months follow-up, when compared to a control group receiving education as usual. This study expands the current literature and scarce research on mindfulness in primary aged children, particularly in the UK context. It informs future research directions, well-being and education policy by highlighting that mindfulness-based programs delivered within the school curricula have the potential to promote self-regulation and improve children’s emotional well-being. However, in the context of limited mindfulness research in children, future research will need to replicate these findings, and further establish how mindfulness impacts children across developmental stages. Programs which promote a child’s ability to manage their negative emotions have the potential to protect psychological well-being across the life span, and if delivered in the school context offer an accessible universal approach. Interventions which enhance EF are not only promising in the context of learning and education, but also hold potential for children experiencing cognitive deficits and those at risk of developing self-regulation problems. The potential role of mindfulness as an early and preventive approach in children that targets both cognitive and affective aspects of self-regulation highlights considerable possible benefits for children.

## Conflict of Interest Statement

The authors declare that the research was conducted in the absence of any commercial or financial relationships that could be construed as a potential conflict of interest.

## Abbreviations

EIPP: Early Intervention Prevention Programs
EF: Executive Functioning
